# Depression, anxiety symptoms, and association with household characteristics in adolescent boys and girls from Matiari District, Pakistan: A community-based cross-sectional study

**DOI:** 10.1371/journal.pone.0350609

**Published:** 2026-06-17

**Authors:** Florence Perquier, Susan C. Campisi, Clare Zasowski, Katherine Tombeau Cost, Yaqub Wasan, Sajid B. Soofi, Mina Husain, Daphne Korczak, Suneeta Monga, Peter Szatmari, Zulfiqar A. Bhutta

**Affiliations:** 1 Cundill Centre for Child and Youth Depression, Centre for Addiction and Mental Health, Toronto, Ontario, Canada; 2 Department of Psychiatry, Hospital for Sick Children, Toronto, Ontario, Canada; 3 Nutrition and Dietetics Program, Clinical Public Health Division, Dalla Lana School of Public Health, University of Toronto, Ontario, Canada; 4 Centre for Global Child Health, Hospital for Sick Children, Toronto, Ontario, Canada; 5 Department of Psychology, University of Waterloo, Waterloo, Ontario, Canada; 6 Department of Behavioural Neurosciences and Psychiatry, McMaster University, Hamilton, Ontario, Canada; 7 Centre of Excellence in Women and Child Health, Aga Khan University, Karachi, Pakistan; 8 Department of Psychiatry, Temerty Faculty of Medicine, University of Toronto, Toronto, Ontario, Canada; Kandahar University, Faculty of Medicine, AFGHANISTAN

## Abstract

**Introduction:**

Pakistan has one of the world’s largest adolescent populations, yet evidence on the prevalence and correlates of depressive and anxiety symptoms in adolescents remains limited, particularly in rural settings.

**Objective:**

This study aimed to estimate the prevalence of depressive and anxiety symptoms and examine their associations with household characteristics in a community-based sample of adolescents from the predominantly rural district of Matiari, Pakistan.

**Methods:**

We examined cross-sectional data from 718 girls (9.0–14.9 years) and 678 boys (10.0–15.9 years) participating in the Nash-wo-Numa Study. Trained psychologists administered the Sindhi versions of the Short Mood and Feelings Questionnaire and the Screen for Child Anxiety Related Emotional Disorders to assess adolescents’ depressive and anxiety symptoms. Prevalence estimates and 95% confidence intervals were derived based on validated cut-off scores. Household correlates of depressive and anxiety symptoms were examined in multivariable negative binomial regression models.

**Results:**

Approximately 8% of boys and 10% of girls exhibited clinically-significant depressive symptoms. The prevalence of clinically-significant anxiety symptoms ranged from 6% in boys and 8% in girls for generalized anxiety to 24% in boys and 39% in girls for separation anxiety symptoms. Girls experienced more depressive symptoms, panic/somatic and generalized anxiety symptoms than boys at age 12, more separation anxiety symptoms from age 11 onward, and more social anxiety symptoms from age 12 onward. In both sexes, depressive and anxiety symptoms were higher among adolescents exposed to intimate partner violence against their mothers and to moderate‑to‑severe food insecurity, and were lower among those with a homemaker mother. Among girls, maternal mental well‑being attenuated the association between food insecurity and depressive symptoms.

**Conclusion:**

Depressive and anxiety symptoms are common among adolescents living in Matiari. Adolescents exposed to intimate partner violence against their mother, moderate-to-severe food insecurity, and poor maternal mental health may be at increased risk of depression and anxiety in predominantly rural Pakistan and may benefit from targeted prevention and intervention strategies.

## Introduction

The transition from childhood to adulthood is a time of significant physical, emotional and social changes that can increase susceptibility to mental health problems [1]. According to a recent meta-analysis, by the age of 18, 26% of people have experienced first symptoms of depressive disorders, and 60% have experienced first symptoms of anxiety or fear-related disorders [2]. Longitudinal studies suggest that anxiety and depression in childhood and adolescence can have lasting effects, increasing the risk of adverse outcomes in adulthood, including substance use, poorer physical health and compromised economic and social outcomes [3,4].

Depression and anxiety are prevalent worldwide. Based on pre-pandemic data, prevalence of depressive and anxiety symptoms in low and middle-income (LMICs) has been reported to be broadly comparable to those observed in high-income countries (HICs), although estimates vary widely across LMICs, ranging from 0 to 28% for depressive symptoms and from 8 to 27% for anxiety symptoms [5]. Prevention, detection, and early intervention strategies in children and adolescents may help minimize the burden of these mental health symptoms in adolescence [6]. However, their impact strongly depends on contextual and cultural factors [7]. As most strategies were developed and evaluated in HICs, concerns remain regarding their applicability and effectiveness in LMICs in general and within specific country contexts [7,8].

Data on the prevalence of anxiety and depressive symptoms remains limited in Pakistan [9], where children and adolescents between 9 and 18 years of age still represent 23% of the population [10]. Prior to the COVID-19 pandemic, only three studies had examined the prevalence of depression and anxiety in this age group [11–13], with two being conducted in urban and school-based settings [11,12]. In sixth graders living in Hyderabad, the prevalence of clinically-significant depressive symptoms, assessed using the Children’s Depression Inventory, was estimated to be 14.9% in girls and 19.9% in boys [11]. The prevalence of clinically-significant depressive symptoms was found to be similar (17.2%) in adolescents aged 11–18 living in Rawalpindi, using the Hospital Anxiety and Depression scale [12]. In the same study, the prevalence of clinically-significant anxiety symptoms was 21.4%. Although informative, these results were obtained among urban school-going adolescents and might not apply to the majority of population of children and adolescents living in Pakistan for two main reasons. First, the majority of the Pakistan’s population lives in rural areas (64%) and, second, approximately 20% of children between 6 and 16 years do not attend school [14]. In rural areas, lower levels of school attainment, higher socioeconomic deprivation, greater exposure to food insecurity and more traditional norms, as well as lower access to mental health care might influence the rates of adolescent depression and anxiety, compared to urban settings [15]. To our knowledge, only one study has estimated the prevalence of depression in girls living in rural Pakistan. Among 321 unmarried 16- to 18-year-old girls living in rural Punjab, the prevalence of clinical depression – measured using the Structured Clinical Interview for DSM-IV Disorders – was 4.4%, a rate lower than what was observed in urban areas [13].

The family home environment, encompassing socioeconomic and interpersonal dynamics, has long been recognized as a central microsystem influencing adolescents’ mental health in both HIC and in LMICs [16]. The role of family and household characteristics has been suggested to be particularly pronounced in LMICs, where institutional supports such as school-based mental health services, community programs, and accessible healthcare are often limited or inconsistent [17]. Adolescents from households with higher socioeconomic status have been shown to exhibit reduced risks of depressed mood and anxiety, whereas adverse interpersonal dynamics within the family, such as conflicts or interpersonal violence, are associated with increased risk [17,18]. Prior findings from studies conducted in Pakistan corroborate those associations: low family affluence was found to be associated with higher levels of depressive and anxiety symptoms [12,13], and food insecurity – often considered strong material indicator of poverty – was associated with higher depressive symptoms in girls living in rural Punjab [13]. Regarding interpersonal dynamics, experiencing corporal punishment at home, witnessing interpersonal violence (IPV) against the mother, and family relationship problems were also found to be associated with greater depressive symptoms [11,13].

Some of these household factors can also affect adolescent’s mental health through parental mental health, which is one of the most consistent predictors of adolescents’ emotional and behavioral problems [19]. According to the Family Stress Model, economic hardship and psychosocial stressors within the household increase parental psychological distress, which in turn can undermine parenting quality, thereby heightening the risk of emotional and behavioral problems in adolescents [19]. Accounting for parental mental health is therefore essential to estimate the direct association between household factors and adolescent mental health symptoms, independent of parental psychological distress. Unfortunately, among prior studies conducted in Pakistan, only one adjusted its analysis for mother’s mental health [13].

There is a need to better understand the prevalence and correlates of anxiety and depressive symptoms in adolescents in Pakistan in general, and in rural areas in particular. Matiari is a predominantly rural district in the province of Sindh, located in southeastern Pakistan. To date, no study has estimated the prevalence of depressive and anxiety symptoms among adolescents living in this district, nor examined their association with household characteristics. Such evidence is needed to improve understanding of adolescents’ mental health needs in this setting and to support the development of contextually relevant interventions and policies. To address those gaps, the current study aims to: i) provide new evidence on the prevalence of depression and anxiety symptoms in both sexes in a representative sample of children and adolescents living in the District of Matiari and ii) estimate the associations of household characteristics with depression and anxiety symptoms in this specific population.

## Methods

### Study population

We used data from the *Nash-wo-Numa* study, a cross-sectional study conducted between January 2019 and February 2020 among early adolescents aged 9 to 15.9 years and their birthmothers living in the Matiari District, Pakistan [20]. Matiari is located in the Pakistani province of Sindh, the second most populous province of Pakistan. According to 2017 Census data, more than 130,000 adolescents aged 9 to 15.9 years live in Matiari, making up 17% of the district’s population [21]. Approximately 77% of them reside in rural areas. The vast majority of the population in Matiari speaks Sindhi.

The primary objective of the *Nash-wo-Numa* study was to examine multiple aspects of adolescent growth, development, and well-being. To meet this objective and to account for the impacts of pubertal onset, which begins earlier in girls, age criteria for inclusion were 9.0–14.9 years for girls and 10.0 to 15.9 years for boys. The Nash-wo-Numa research team identified eligible households using a list established during a previous household census conducted among 53,000 households covered by Female Health Workers in the catchment population of 26 health facilities in Matiari District [22]. They selected a representative sample of households using computer-assisted random sampling based on sex, age, and the number of occupants in each household [20]. If multiple adolescents from the same household were eligible, only one was randomly selected.

The Nash-wo-Numa Study staff visited a total of 1,873 eligible households. The level of urbanization (rural, peri-urban, or urban) for each household was determined based on household address and census classification. Participants outside the eligible age range (9 to 15.9 years) were not included (n = 151), nor were adolescents whose mothers were not available, did not consent to participate, or were cognitively unable to be interviewed (n = 9), adolescents who suffered from a chronic or genetic illness known to impact growth (e.g., congenital heart disease, diabetes, or Down syndrome) and girls who were or had been pregnant (n = 52). During the data management process, we identified 26 participants whose age did not meet the sex-specific age criteria; those were also excluded. For the purpose of the present study, we removed 239 participants with no data on the depressive and anxiety symptoms scales, leaving a final sample of 1,396 participants.

Both informed written consent from the parent or legal guardian and written assent from adolescents were obtained. Ethics approval for the original study was granted by the Ethics Review Committee at the Aga Khan University, Karachi, Pakistan (5251-WCH-ERC-18) and Research Ethics Board at SickKids Hospital, Toronto, Canada (1000060684). Ethics approval for the current study (secondary use of data) was received from the Centre for Addiction and Mental Health Research Ethics Committee (107/2021). Data were accessed on December 11, 2021. Authors had no access to information that could identify individual participants during or after data collection. Additional information regarding the ethical, cultural, and scientific considerations specific to inclusivity in global research is included in the Supporting Information (S8 Checklist)”.

### Procedure

Staff members coordinated appointments and transportation to a field-based clinic. Participants and their mother were interviewed together, with a chaperone present when necessary, using structured questionnaires intended for either mothers (six modules) or adolescents (nine modules). Study psychologists administered mental health measures.

All questionnaires were translated from English into Sindhi and reviewed by psychologists and staff members fluent in both languages. They were pilot-tested in the community before being used in the study. Further details regarding the study protocol can be found elsewhere [23].

### Outcomes

Scales and subscales characteristics (number of items, scores range, Cronbach’s alpha and threshold used to define clinically-relevant symptoms) are summarized in Table 1.

**Table 1:** Characteristics of scales and subscales used to assess mental health outcomes.

Scales and subscales	Number of items	Cronbach’s alpha		Score range	Threshold used to define clinically-significant symptoms
		Boys	Girls		
SMFQ	13	0.85	0.86	0-26	≥8 [24]
SCARED – Panic/somatic	13	0.89	0.91	0-26	≥7 [29]
SCARED – Generalized anxiety	9	0.74	0.76	0-18	≥9 [29]
SCARED – Separation anxiety	8	0.67	0.71	0-16	≥5 [29]
SCARED – Social anxiety	7	0.79	0.81	0-14	≥8 [29]
SCARED – School avoidance	4	0.60	0.58	0-8	≥3 [29]
SCARED – Total*	37	0.91	0.93	0-74	No threshold

* Total score computed using the items of the panic/somatic, generalized anxiety, separation anxiety and social anxiety subscales.

**Depressive symptoms** were self-reported by adolescents using the Short Mood and Feelings Questionnaire (SMFQ), which assesses affective and cognitive symptoms of depression experienced by children and adolescents [24]. Participants were asked to rate how they had been feeling or acting recently (e.g., “I did not enjoy anything at all” or “I felt I was a bad person”), with each item being rated using a 3-point Likert Scale (“not true” = 0, “sometimes” = 1 or “true” = 2). The SMFQ has been validated in community-based samples [25–27]. In the Nash-wo-Numa study, the SMFQ demonstrated strong unidimensionality (Comparative Fit Index = 0.97 in a confirmatory factor analysis of a single factor model) [28], and good internal consistency (see Table 1). In accordance with the original validation study, a total score of 8 or more was considered as indicative of clinically-significant depressive symptoms [24].

**Anxiety symptoms** were assessed based on information provided by adolescents using the Screen for Child Anxiety Related Emotional Disorders (SCARED), initially developed by Birmaher et al. [29]. Five SCARED subscales screen for panic disorder or significant somatic symptoms, generalized anxiety disorder, separation anxiety disorder), social anxiety disorder and – among adolescents attending school – school avoidance. The presence of each symptom over the past three months (e.g., “I am nervous” or “I worry about what is going to happen in the future”) was rated as 0 (Not true or hardly ever true), 1 (Sometimes true) or 2 (True or often true). The psychometric properties of the SCARED have been validated across different countries, including LMICs such as South Africa and China [30]. For each subscales, we used international thresholds previously suggested to indicate the presence of clinically-significant anxiety disorders [29]. Although the psychometric properties of the SCARED were satisfactory in LMICs [31,32], its international thresholds have not been consistently validated, with studies reporting different optimal cut-offs values [33]. A total anxiety score was computed using the items of the panic/somatic, generalized anxiety, separation anxiety and social anxiety subscales (37 items). Items from the school avoidance subscale were not included, as they were administered only to the subsample of adolescents who attended school. A similar score was employed in a study conducted among adolescents from a rural community in India [34]. To date, no universally accepted cut-off has been established for this version of the total SCARED score.

### Household characteristics

Household characteristics included socioeconomic factors commonly examined in relation to adolescent depression and anxiety in LMICs, including mother’s marital status, mother’s and partner’s employment and school attendance, household wealth, and food insecurity [17,18]. IPV has been reported as a key correlate of adolescent mental health outcomes in LMIC settings [13,35] and was used to capture interpersonal dynamics within the household.

**Mother marital status** was declared by the mother herself and classified as ‘married’ or ‘widowed, divorced or separated’.

**Mother’s and partner’s working school attendance.** Mother reported whether she and her partner had ever attended school.

**Mother’s and partner’s working status.** Mothers were asked about their own occupation as well as their partner’s. Partner’s occupation was categorized as ‘manual labour’ (skilled and unskilled and agriculture), ‘non manual labour’ (i.e., sales and services, professional jobs), and ‘unemployed’. Because most mothers reported being housewives, we created a binary variable to distinguish homemaker mothers from those who had an occupation.

**Wealth Index**. The Wealth Index is a composite indicator of a household’s cumulative living standard, used as a proxy for socioeconomic position [36]. Information about housing characteristics, ownership of house/land, number of rooms, fuel used for cooking, source of drinking water, type of sanitation, assets and livestock were reported by mothers. The Wealth index was created using principal component analysis on these variables as recommended by Filmer and Pritchett [36]. The first component obtained was considered as a proxy indicator of the level of wealth at the household level and was split into five wealth quintiles (poorest, poor, middle, richer and richest quartiles). The poorest and poor categories were then combined into a single category “poor”, and the middle, richer and richest wealth quintiles were grouped into “non-poor”.

**Food insecurity**. Food insecurity is described as a direct consequence of insufficient economic access to food and is closely tied to income poverty, especially in rural areas [36]. Mothers were administered the Food Insecurity Experience Scale (FIES), which consists of eight yes/no (1/0) questions referring to the experience of food insecurity at the household level over the last 12 months (i.e., “Was there a time when your household ran out of food because of a lack of money or other resources?”) [37]. The internal consistency of the scale was very good in both boys (α = 0.96) and girls (α = 0.92). The FIES score was categorized as follows: 0–3 = food secure/mild food secure, and 4–8 = moderate to severe food insecure in accordance with the Food and Agriculture Organization of the United Nations’ guidelines [38].

**Mother’s exposure to Intimate partner violence (IPV)**. Mothers reported intimate partner violence using three subscales of the Conflict Tactics Scale short form (CTS2S) [39], each including 2 items: psychological aggression (“My partner insulted or swore or shouted or yelled at me”; “My partner destroyed something belonging to me or threatened to hit me”), physical assault (“My partner pushed, shoved, or slapped me”; “My partner punched, or kicked, or beat me up”) and injuries due to partner violence (“I had a sprain, bruise, or small cut, or felt pain the next day because of a fight with my partner”; “I went to see a doctor or needed to see a doctor because of a fight with my partner”). IPV was coded as present if any of the six behaviours occurred in the past year.

Religion, measured at the household level, was used to describe the sample of boys and girls included in our sample, but was not considered as an explanatory variable in the present study, as our aim was to address factors that could be modified by public health or social interventions.

### Adolescent’s characteristics

Date of birth and sex assigned at birth were reported by mothers.

**Age** was calculated as the number of years between the date of birth reported by the mother and the date of the interview at the field-based clinic.

**School attendance** was assessed by asking adolescents whether they were currently attending school at the time of the survey.

### Mother’s mental well-being

Trained psychologists administered the 14-item Warwick-Edinburgh Mental Wellbeing Scale (WEMWBS) to mothers to assess the feelings and thoughts they experienced over the last two weeks (e.g., “I’ve been feeling good about myself”) [40]. Each item was rated from 1 (none of the time) to 5 (all of the time), with total sum scores ranging from 14 to 70. Higher scores indicated a higher level of mental wellbeing. The WEMWBS demonstrated good internal consistency in our sample (α = 0.91 in both boys and girls).

### Analysis

Analyses were stratified by sex to address the evidence gap regarding boys’ mental health, and because age ranges were different for boys and girls. Prevalence estimates were derived based on international threshold for each instrument and its subscales and confidence intervals were obtained using the Wilson score method. Differences in prevalence estimates according to sex were compared within overlapping age ranges using chi-square tests.

Depression and anxiety scores were right-skewed (i.e., more scores at the lower end of the scale). Correlations between scores and between covariates were examined using Spearman rho.

We estimated the association of household characteristics independently of adolescent characteristics (age and school attendance) and mother’s well-being. Negative binomial regression was preferred to Poisson regression because of significant over-dispersion (likelihood ratio test of the dispersion parameter, p < .001 in all models). Associations were described using incident rate ratios (IRR). The IRR corresponds to the ratio of the rates of symptoms in two different groups. The association of each household characteristic with depressive and anxiety symptoms was first estimated in models adjusted for age and school attendance (in models M1). All the adolescent and household characteristics were then introduced simultaneously in a full model (M2). We added mother’s mental well-being in a third model (M3). When a covariate was significant in M2 but not significant in M3, we tested for potential interactions between that covariate and mother’s well-being in predicting the outcome.

All statistical analyses were performed using STATA version MP/16 (Stata Corp., College Station, Texas, USA). A p-value less than 0.05 was set to be statistically significant.

## Results

### Participants

The characteristics of the 678 boys (mean age = 12.9; SD = 1.6) and the 718 girls (mean age = 12.0; SD = 1.7) included in the study are described in Table 2. Nearly three quarters of boys, but only half of the girls, attended school. More than 90% of mothers were married and were living with their partner. The vast majority of mothers’ partners were working in manual labour and agriculture. Mothers of girls were younger and more likely to have attended school. A greater proportion of girls than boys lived in Muslim families, whereas boys were more likely than girls to reside in rural areas.

**Table 2:** Characteristics of participants.

	Boys (N = 678)		Girls (N = 718)		
	n	%	n	%	p
**Participants characteristics**					
Age in years, mean[SD] ^#^	12.9	[1.6]	12.0	[1.7]	**^w^ < .001**
School attendance	508	74.9	375	52.2	**<.001**
**Household characteristics**					
Living in a rural area	551	81.3	536	74.7	**.003**
Religion					
Muslim	548	80.8	619	86.1	**.007**
Hindu	130	19.2	99	13.8	
Wealth Index (quintiles)					
Poor (Q1, Q2)	276	40.7	287	40.0	.779
Non Poor (Q3, Q4, Q5)	402	59.3	431	60.0	
Moderate to severe food insecure (FIES≥4)	165	24.3	167	23.3	.637
Mothers’ characteristics					
Mother’s age in years, mean[SD]	40.2	[5.9]	39.2	[5.8]	**^w^ < .001**
Mother’s marital status					
Married	633	93.4	663	92.3	.459
Widowed, divorced or separated	45	6.6	55	7.7	
Mother’s school attendance	104	15.3	155	21.6	**.003**
Mother’s working status					
Working	274	40.4	273	38.0	.360
Homemaker	404	59.6	445	62.0	
Intimate partner violence against mother					
No	212	31.4	262	36.5	.076
Yes	419	61.8	401	55.9	
Missing	47	6.9	55	7.7	
Mother’s mental health well-being (WEMWBS), mean[SD]	51.9	[9.7]	51.8	[10.1]	^w^.810
Partners’ characteristics					
Partner living at home	633	93.4	669	93.2	.889
Partner’s school attendance	341	50.3	397	55.3	.062
Partner’s occupation					
Manual work, agriculture	555	81.9	580	80.8	.854
Sales, service, professional, others	116	17.1	131	18.3	
Unemployed	7	1.0	7	1.0	

# 5 participants with missing data; ^w^ p-value from the Wilcoxon rank sum test; SD, standard deviation; Q, Quintile; FIES, Food Insecurity Experience Scale; WEMWBS, Warwick-Edinburgh Mental Wellbeing Scale.

Compared to participants included in the analysis, those excluded because of missing mental health data (n = 239) were significantly older, their mother’s partner was less likely to work in manual labour and they were less exposed to food insecurity (see S1 Table).

### Prevalence

The mean SMFQ score was 2.3 (SD = 3.2; min = 0; max = 21) in boys and 2.7 (SD = 3.5; min = 0; max = 24) in girls. The prevalence of clinically-significant depressive symptoms was 8.1% (95%CI 6.3–10.4) in boys and 10.2% (95%CI 8.2–12.6) in girls (S2 Table).

Boys had a mean anxiety symptom score of 12.5 (SD = 9.9; min = 0; max = 56). The most prevalent clinically-significant symptoms were symptoms of separation anxiety (23.9%; 95% CI 20.8–27.3), followed by symptoms of panic/somatic disorder (13.3%; 95%CI 10.9–16.0), social anxiety (11.2%; 95%CI 9.1–13.8), and generalized anxiety disorder (5.8%; 95%CI 4.2–7.8). In boys attending school (n = 508), the prevalence of school avoidance was 15.8% (95%CI 12.8–19.1).

Girls had a mean anxiety symptom score of 16.7 (SD = 11.8; min = 0; max = 72). The prevalence of clinically-significant symptoms of separation anxiety disorder was, again, the highest (39.1%; 95%CI 35.6–42.8), followed by symptoms of social anxiety disorder (22.3%; 95%CI 19.4–25.5), panic or significant somatic disorders (17.1%, 95%CI 14.6–20.1), and generalized anxiety disorder (7.7%; 95%CI 5.9–9.8). The prevalence of school avoidance was 15.5% (95%CI 12.2–19.5) in the subsample of 375 girls who attended school.

Age-specific prevalence estimates were compared according to sex from age 10 to age 14. Significantly more girls than boys presented clinically-significant symptoms of depression, panic or somatic disorder and generalized anxiety disorder at age 12 (S2 Table). Girls were more likely to experience clinically-significant symptoms of separation anxiety at age 11, 12, 13 and 14, and to experience clinically-significant symptoms of social anxiety at age 12, 13 and 14. No sex difference was found in the prevalence of school avoidance.

### Association of symptom scores with household characteristics

Depression and anxiety symptom scores were moderately correlated (Spearman ρ = 0.60; p < .001) and correlations between explanatory variables ranged from negligible to moderate (S3 Table).

Results of the final multivariable regression models describing the association between household characteristics and symptom scores are presented in Table 3 and 4. Results obtained in simple (M1) and intermediate multi-adjusted models (M2) are available in S4-S7 Tables.

**Table 3:** Association of household characteristics with depressive and anxiety symptoms in adolescent boys living in Matiari, Pakistan (n = 678).

	Depressive symptoms			Anxiety symptoms		
	IRR	[95%CI]	p	IRR	[95%CI]	p
**Participants characteristics**						
Age in years	1.044	0.982-1.110	.171	0.989	0.955-1.024	.525
School attendance						
No	0.881	0.700-1.109	.280	**0.845**	**0.740-0.966**	**.014**
Yes	Ref			Ref		
**Household characteristics**						
Living Area						
Urban	Ref			Ref		
Rural	0.822	0.648-1.043	.107	0.960	0.835-1.103	.565
Mother’s marital status						
Married	Ref			Ref		
Widowed, divorced or separated	1.042	0.357-3.042	.940	0.634	0.340-1.181	.151
Mother’s working status						
Working	Ref			Ref		
Homemaker	**0.744**	**0.611-0.906**	**.003**	**0.891**	**0.794-0.999**	**.047**
Mother school attendance						
No	0.928	0.701-1.227	.600	1.075	0.916-1.261	.376
Yes	Ref			Ref		
Partner’s occupation						
Manual labour, agriculture	Ref			Ref		
Sales, service, professional, others	1.258	0.951-1.663	.108	**1.176**	**1.005-1.376**	**.043**
Unemployed	1.814	0.799-4.121	.155	1.106	0.656-1.864	.706
Partner’s school attendance						
No	0.985	0.803-1.208	.881	1.007	0.895-1.133	.906
Yes	Ref			Ref		
Intimate partner violence						
No	Ref			Ref		
Yes	**1.614**	**1.301-2.003**	**<.001**	**1.198**	**1.062-1.350**	**.003**
Missing	1.143	0.396-3.299	.804	1.313	0.712-2.424	.383
Wealth Index (quintiles)						
Poor (Q1, Q2)	0.871	0.710-1.070	.188	1.000	0.890-1.124	.995
Non Poor (Q3, Q4, Q5)	Ref			Ref		
Food insecurity (FIES)						
Food secure/Mild food insecure	Ref			Ref		
Moderate to severe food insecure	**1.454**	**1.158-1.826**	**.001**	**1.224**	**1.070-1.401**	**.003**
Mother’s mental health well-being (WEMWBS score)	**0.950**	**0.940-0.959**	**<.001**	**0.970**	**0.964-0.976**	**<.001**

IRR, Incidence Rate Ratio; CI, Confidence Interval; FIES, Food Insecurity Experience Scale; WEMWBS, Warwick-Edinburgh Mental Wellbeing Scale.

**Table 4:** Association of household characteristics with depressive and anxiety symptoms in adolescent girls living in Matiari, Pakistan (n = 718).

	Depressive symptoms			Anxiety symptoms		
	IRR	[95%CI]	p	IRR	[95%CI]	p
**Participants characteristics**						
Age in years	1.014	0.959-1.071	.624	0.990	0.961-1.020	.498
School attendance						
No	1.162	0.955-1.414	.134	1.104	0.991-1.230	.073
Yes	Ref			Ref		
**Household characteristics**						
Living Area						
Urban	Ref			Ref		
Rural	0.859	0.708-1.042	.123	**0.882**	**0.792-0.982**	**.022**
Mother’s marital status						
Married	Ref			Ref		
Widowed, divorced or separated	1.399	0.257-7.624	.698	0.856	0.349-2.102	.735
Mother’s working status						
Working	Ref			Ref		
Homemaker	**0.716**	**0.598-0.858**	**<.001**	**0.897**	**0.811-0.992**	**.034**
Mother school attendance						
No	0.859	0.685-1.076	.185	0.985	0.871-1.114	.813
Yes	Ref			Ref		
Partner’s occupation						
Manual labour, agriculture	Ref			Ref		
Sales, service, professional, others	0.970	0.767-1.228	.803	1.040	0.916-1.180	.546
Unemployed	0.838	0.357-1.966	.685	0.945	0.590-1.513	.813
Partner’s school attendance						
No	0.989	0.822-1.189	.903	1.011	0.914-1.119	.829
Yes	Ref			Ref		
Intimate partner violence						
No	Ref			Ref		
Yes	**1.421**	**1.181-1.709**	**<.001**	**1.176**	**1.064-1.299**	**.001**
Missing	0.784	0.143-4.310	.780	1.118	0.455-2.747	.808
Wealth Index (quintiles)						
Poor (Q1, Q2)	0.935	0.783-1.115	.452	0.957	0.869-1.055	0.376
Non Poor (Q3, Q4, Q5)	Ref			Ref		
Food insecurity (FIES)						
Food secure/Mild food insecure	Ref			Ref		
Moderate to severe food insecure	**3.040**	**1.195-7.735**	**.020**	**1.138**	**1.011-1.280**	**.032**
Mother’s mental health well-being (WEMWBS score)	**0.962**	**0.953-0.972**	**<.001**	**0.975**	**0.970-0.980**	**<.001**
Mother’s mental health well-being X Food insecurity	**0.978**	**0.959-0.997**	**.024**			

IRR, Incidence Rate Ratio; CI, Confidence Interval; FIES, Food Insecurity Experience Scale; WEMWBS, Warwick-Edinburgh Mental Wellbeing Scale; X, Interaction.

In boys, lower levels of depressive and anxiety symptoms were found in boys whose mother was a homemaker (respectively IRR = 0.74; IC95% = 0.61–0.91; p = .003 and IRR = 0.89; IC95% = 0.79–1.00; p = .047), compared to boys whose mother had a professional activity (Table 3). Higher levels of depressive and anxiety symptoms were found in boys exposed to intimate partner violence against their mother (IRR = 1.61; IC95% = 1.30–2.00; p < .001 and IRR = 1.20; IC95% = 1.06–1.35; p = .003) and to food insecurity (IRR = 1.45; IC95% = 1.16–1.83; p = .001 and IRR = 1.22; IC95% = 1.07–1.40; p = .003).

**Fig 1:**
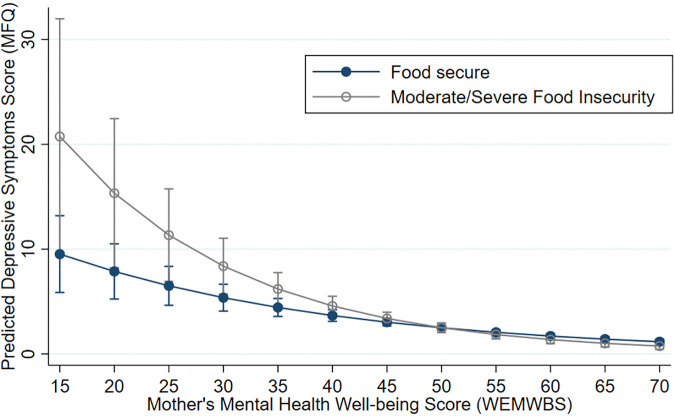
Predicted depressive symptoms score according to mother’s mental health well-being score and food insecurity in girls living in Matiari, Pakistan (n = 718).

In girls, living in a rural area was associated with decreased symptoms of anxiety (IRR = 0.88; IC95% = 0.79–0.98; p = 0.022) (Table 4). As found in boys, levels of depressive and anxiety symptoms decreased in girls whose mother was a homemaker (respectively IRR = 0.72; IC95% = 0.60–0.86; p < .001 and IRR = 0.90; IC95% = 0.81–0.99; p = .034) and increased with exposure to intimate partner violence (IRR = 1.42; IC95% = 1.18–1.71; p < .001 and IRR = 1.18; IC95% = 1.06–1.30; p = .001). The significant positive relationship between food insecurity and depressive symptoms, originally found in model M2 (p = .008, see S6 Table), was no longer significant after adjustment for mother’s mental health well-being (in model M3, p = 0.553). A significant negative interaction was found between food insecurity and mother mental health (IRR = 0.98; IC95% = 0.66–1.00; p = 0.024) and was included in the final model predicting depressive symptoms. Moderate to severe food insecurity was associated with higher anxiety symptoms (IRR = 1.14; IC95% = 1.01–1.28; p < .001). As shown in Fig 1, among girls, the association between moderate-to-severe food insecurity and depressive symptoms was stronger when mothers had low mental health well-being scores, and weakened as mother’s mental well-being score increased. No significant interaction was found between food insecurity and mother’s mental health well-being when predicting anxiety symptoms (p = .127).

## Discussion

Our cross-sectional study provides new evidence regarding the prevalence and household correlates of both depression and anxiety symptoms among a representative sample of adolescent boys and girls living in the district of Matiari, Pakistan. It is the largest study conducted in a mainly rural setting and the first to include boys and a measure of anxiety symptoms. Unlike previous school-based studies conducted in Pakistan, it includes participants who did not attend school, who are typically under-represented in the existing literature.

The prevalence of clinically-significant depressive symptoms was 8.1% in boys and 10.2% in girls, which is lower than previous estimates obtained in Pakistan (ranging between 15 and 20%) or in other Asian countries [41]. Such differences might relate to differences in study settings, as previous studies were carried out among school-going adolescents living in urban areas only [11,12]. The inclusion of participants living in rural areas (more than 75% of participants in our study) could also explain our findings. Research on rural-urban differences in adolescent mental health in LMICs is scarce, but some recent studies conducted in rural adolescents suggest that they experience fewer depressive symptoms compared to their urban counterparts [42–44]. Living in an urban area may be associated with greater exposure to social and economic inequalities, violence and deleterious environmental conditions (pollution, lack of green spaces), that are risk factors for depression [45]. Urban settings in Pakistan are also shaped by high levels of internal migration, particularly rural-to-urban movement driven by employment and education opportunities [46]. In LMICs, migration processes and post-migration adaptation have been shown to increase psychological distress in adolescents through disruption of social networks, reduced familial cohesion, and increased economic insecurity [47,48]. In contrast, the rural setting of Matiari may offer more stable household and community structures, potentially acting as a protective factor. In addition to urban-rural differences, our results could be explained by the fact that our participants were younger than those included in previous studies. As depression rates increase sharply from early to middle adolescence [1], higher estimates would be expected in adolescents older than 15 years old. Lower rates of depressive symptoms may also be due to underreporting, as interviews were not conducted in private.

The prevalence estimates of anxiety disorders were higher than those usually observed in HICs and in other studies conducted in LMICs. In particular, separation anxiety disorder was found to be highly prevalent in our sample, especially among girls (39.1%). The SCARED instrument offers the advantage of assessing multiple anxiety disorder domains simultaneously, whereas most measures used in previous studies, such as the HADS, primarily focus on general anxiety. This difference may contribute to the variability in reported prevalence estimates across studies. This pattern may also reflect measurement and cultural interpretation issues rather than true differences in disorder prevalence. Indeed, SCARED thresholds were mainly developed and validated in HICs, but are not consistently validated in LMIC contexts [33]. In rural India, the SCARED assessment also yielded high separation anxiety scores among adolescents aged 11–19 years [34]. However, the international SCARED cut-off thresholds did not demonstrate optimal diagnostic accuracy when compared to DSM-IV-TR diagnoses derived from the K-SADS-PL semi-structured interview and tended to overestimate the prevalence of separation anxiety disorders [49]. Cultural context is particularly relevant: items such as fear of being alone at home – endorsed by more than 40% of participants in our study – may reflect normative living arrangements in rural Pakistan, where multigenerational households are common and adolescents are rarely alone, rather than pathological anxiety. Both separation anxiety and social anxiety symptoms have been shown to depend on social norms and expectations, and to differ across cultures [50]. The SCARED subscales’ internal consistency was acceptable in the present work. Unfortunately, we were not able to examine the diagnostic accuracy of the international SCARED thresholds used to define anxiety disorders. We recommend that future studies exercise caution when applying SCARED international thresholds in LMICs, as more evidence is needed on the optimal cut-off values to be used in specific social and cultural contexts.

Sex differences observed in our study, with higher depressive and anxiety symptoms among girls, are consistent with global evidence [8,51–53]. The most pronounced differences emerged at age 12, which corresponds to the median age of puberty in this sample [54]. These differences likely reflect a combination of both universal and context-specific mechanisms. Biological factors, including hormonal and physiological changes during puberty, may increase vulnerability to adverse mental health outcomes among females compared with males [55,56]. Alongside biological changes, social explanations highlight the role of gender norms, which can constrain opportunities and shape expectations, often disproportionately affecting girls [57]. In Pakistan, as an Islamic republic, and especially in rural areas, these social dynamics may be intensified by more constraints on girls’ autonomy in areas such as education, mobility, and life choices.

Despite differences according to sex, many of the same household characteristics were associated with anxiety and depressive symptoms in both boys and girls. Among these, adolescents with homemaker mothers had lower levels of both depressive and anxiety symptoms. This finding is consistent with results from previous research conducted in other LMICs, suggesting that maternal unemployment tends to improve mental health outcomes in children and adolescents [58,59]. In our sample, the majority (more than 60%) of mothers were homemakers, reflecting traditional household arrangements in Pakistan, where women are often the primary caregivers, while men are more frequently engaged in income-generating activities outside the home. These findings may reflect greater maternal availability for caregiving in a context where most women are homemakers, which could benefit adolescent mental health [60]. However, this association may also reflect broader socioeconomic dynamics. In this setting, maternal employment may sometimes be driven by financial necessity, potentially reflecting greater social and economic vulnerability, which may in turn influence their children’s mental health. Although we adjusted our analyses on many socioeconomic factors to limit confounding bias, we cannot rule out the effect of unmeasured socioeconomic factors that may have influenced this relationship.

Our results also supported an independent association of food insecurity with higher levels of anxiety symptoms in both boys and girls, and with higher levels of depressive symptoms in boys. Food insecurity has been shown to be associated with poorer adolescents’ mental health in LMICs [11,61], as well as in HICs [62,63]. The association of food insecurity with mental health might be explained by the poor quality of the diet (and the decreased intake of beneficial macronutrients), by the effect of malnutrition on body weight (stunting or obesity), and by higher level of psychosocial stress, due to the feeling of deprivation and food supply related concerns [64]. However, in girls, we found that poor mother’s mental health exacerbated the association of food insecurity with depressive symptoms, suggesting that the relationship between food insecurity and girls’ depressive symptoms is unlikely to follow a simple linear pathway. Indeed, food insecurity is closely linked to parental mental health, which can mediate and amplify its effect on adolescents through parenting quality and emotional availability [65]. In US adolescents aged 10–14 years, food insecurity had both direct and indirect effects on adolescents’ behaviour problems [19]. Higher levels of caregiver depressive symptoms and poorer quality of the adolescent–caregiver relationship were found to mediate the indirect association [19]. Conversely, poor mother’s mental health may also contribute to food insecurity through reduced participation in economic activities, diminished caregiving capacity, and suboptimal child nutrition practices, thereby further affecting adolescents’ mental health [66]. Given the cross‑sectional nature of our study, we could not determine the temporal ordering of these processes, and future longitudinal studies are needed to clarify the complexity of these pathways, especially in girls.

Exposure to IPV against mothers was associated with higher risk of depressive and anxiety symptoms in both boys and girls. Not only IPV against women affects mothers’ physical, reproductive, and mental health, but mounting evidence suggest that it is associated with the risk of depression and anxiety in their adolescent offspring [11,67]. Meta-analytic reviews demonstrate that exposure to IPV increases a child’s risk of further internalizing symptoms and externalizing behaviour [68,69], and favours the development of an insecure attachment to their primary caregiver [70]. The association observed in our study remained after adjustment for socioeconomic characteristics and mother mental health. However, adolescents exposed to IPV may also be at increased risk of abuse and neglect and may engage in more risky behaviours – dimensions that were not collected in our study but are also positively associated with higher depression and anxiety symptoms [71]. Although IPV against women occurs in all countries, the prevalence of emotional and physical violence IPV is especially high in Pakistan (respectively, 36.4% and 18.4% in married women) [72], and might affect multiple generations, as it is common for grandparents, parents and children to live in the same household [11].

### Strengths and limitations

Despite the rigorous selection of participants being a key strength of our study, a selection bias may still have occurred, as those excluded due to the absence of mental health data were slightly older and less exposed to food insecurity. This may have affected the prevalence estimates, but does not undermine the validity of the associations between household characteristics and depressive and anxiety symptoms.

Our large sample size allowed us to robustly examine the independent associations of multiple household characteristics with depression and anxiety symptoms. But small cell counts in some categories (such as mother’s marital status or partner’s unemployment) may have affected the precision of our estimates and the significance of our results. For these variables, limited statistical power may have biased our results toward non-significance. Moreover, the selection of household characteristics was constrained by the available data, with many variables centered around the mother. Some of these variables, such as the mother’s “homemaker” status, may reflect diverse household arrangements or caregiving practices, which were not measured in our study. The characteristics included may represent only part of the broader household context and should be interpreted alongside their limitations, including the lack of comprehensive measures of caregiving practices and more paternal characteristics.

Due to cultural sensitivities, the child and the mother were interviewed together, sometimes in the presence of a chaperone. While this approach improved the feasibility of the study, it may have increased social desirability bias. This could have led to under-reporting of anxiety and depressive symptoms, as well as other sensitive information, such as IPV. If so, such misclassification could have resulted in the underestimation of the prevalence estimates and of the associations observed.

Psychometric interviews in Sindhi by trained psychologists were employed to reduce language and comprehension barriers and the scales used demonstrated acceptable to excellent reliability in our sample [28]. Unfortunately, clinical measures of depression and anxiety disorders were not collected, which prevented us to assess the validity of international thresholds in our population. Although psychometric assessment tools cannot be used to diagnose clinical disorders, they are useful to quantify the severity of symptoms, and to screen for ‘probable’ cases of depression. Their use as proxies for clinical diagnosis in prevalence studies has important advantages for estimating population’s needs and enabling comparisons, but they have been criticized for having a high chance of false-positive results and thus, for overestimating the burden of diseases [73].

Due to our cross-sectional design, the associations observed should not be interpreted as causal. Longitudinal studies are needed to better understand the temporal relationships between household characteristics, mother’s mental health and anxiety and depression in adolescents. To date, no such study has been conducted in Pakistan.

Finally, the generalizability of our findings to other locations and settings (particularly urban areas) should be considered with caution. The data were collected prior to COVID and the 2022 floods and may not reflect the current situation. However, our estimates may serve as a useful reference for understanding how these events have subsequently impacted adolescents’ mental health, particularly in the district of Matiari.

## Conclusion

Our results reaffirm the importance of addressing mental health needs in Pakistani adolescents without limiting research to urban samples. Investments in mental health can have an estimated four-fold return [74]. Despite recent progress, less than one percent of Pakistan’s annual health budget is allocated to mental health [75] and less than 5 qualified child and adolescent psychiatrists exist in the country of more than 220 million people [76]. The identification of population subgroups at higher risk of depressive and anxiety symptoms is crucial to inform future care and intervention strategies that would benefit boys and girls. Based on our findings, intimate partner violence against mothers, moderate to severe food insecurity and maternal mental health might be priority targets to reduce the burden of depressive and anxiety symptoms in early adolescents living in rural Pakistan. Results from future population-based studies conducted in other rural locations, and using a longitudinal design, are needed to corroborate our conclusions.

## Supporting information

S1 Table Characteristics of participants included in and excluded from the analysis. (DOCX)

S2 Table Prevalence estimates in boys and girls, by age. (DOCX)

S3 Table Spearman correlations between covariates in boys and girls. (DOCX)

S4 Table Association of household characteristics with depressive symptoms in boys living in Matiari, Pakistan (n = 678). (DOCX)

S5 Table Association of household characteristics with anxiety symptoms in boys living in Matiari, Pakistan (n = 678). (DOCX)

S6 Table Association of household characteristics with depressive symptoms in girls living in Matiari, Pakistan (n = 718). (DOCX)

S7 Table Association of household characteristics with anxiety symptoms in girls living in Matiari, Pakistan (n = 718). (DOCX)

S8 Checklist Inclusivity in global research questionnaire. (PDF)
