# Supplementary material for: Depression, anxiety symptoms, and association with household characteristics in adolescent boys and girls from Matiari District, Pakistan: A community-based cross-sectional study
Source: PLoS One. 2026 Jun 17;21(6):e0350609. doi: 10.1371/journal.pone.0350609 (PMC13274832; doi:10.1371/journal.pone.0350609)
Supplement: S1 Table — (DOCX) [file pone.0350609.s001.docx]

**S1 Table. Characteristics of participants included in and excluded from the analysis.**

|  | Included (N=1396) | | Excluded (N=239) | |  |
| --- | --- | --- | --- | --- | --- |
|  | n | % | n | % | p |
| **Sex** |  |  |  |  |  |
| Male | 678 | 48.57 | 128 | 53.56 | .154 |
| Female | 718 | 51.43 | 111 | 46.44 |  |
| **Age (girls), n=829** |  |  |  |  |  |
| 09.0-10.9 years | 244 | 33.98 | 38 | 34.23 | **.002** |
| 11.0-12.9 years | 243 | 33.84 | 21 | 18.92 |  |
| 13.0-14.9 years | 231 | 32.17 | 52 | 46.85 |  |
| **Age (boys), n=806** |  |  |  |  |  |
| 10.0-11.9 years | 224 | 33.04 | 33 | 25.78 | **.050** |
| 12.0-13.9 years | 232 | 34.22 | 39 | 30.47 |  |
| 14.0-15.9 years | 222 | 32.74 | 56 | 43.75 |  |
| **Mother's marital status, n=1,586** |  |  |  |  |  |
| Married | 1296 | 92.84 | 176 | 92.63 | .918 |
| Not Married | 100 | 7.16 | 14 | 7.37 |  |
| **Living area, n=1,635** |  |  |  |  |  |
| Urban | 309 | 22.13 | 63 | 26.36 | .150 |
| Rural | 1087 | 77.87 | 176 | 73.64 |  |
| **Wealth index, n=1,587** |  |  |  |  |  |
| Richest | 833 | 59.67 | 117 | 61.26 | .675 |
| Poorest | 563 | 40.33 | 74 | 38.74 |  |
| **Mother homemaker, n=1,586** |  |  |  |  |  |
| No | 547 | 39.18 | 66 | 34.74 | .238 |
| Yes | 849 | 60.82 | 124 | 65.26 |  |
| **Partner’s occupation, n=1586** |  |  |  |  |  |
| Manual labor, agriculture | 1135 | 81.3 | 128 | 67.37 | **<.001** |
| Sales, service, professional, others | 247 | 17.69 | 68 | 31.58 |  |
| Unemployed | 14 | 1 | 2 | 1.05 |  |
| **Mother's school attendance, n=1,635** |  |  |  |  |  |
| Yes | 1137 | 81.45 | 193 | 80.75 | .799 |
| No | 259 | 18.55 | 46 | 19.25 |  |
| **Partner's school attendance, n=1,635** |  |  |  |  |  |
| Yes | 658 | 47.13 | 119 | 49.79 | .447 |
| No | 738 | 52.87 | 120 | 50.21 |  |
| **Food insecurity, n=1,586** |  |  |  |  |  |
| No | 1064 | 76.22 | 170 | 89.47 | **<.001** |
| Yes | 332 | 23.78 | 20 | 10.53 |  |
