# Supplementary material for: Depression, anxiety symptoms, and association with household characteristics in adolescent boys and girls from Matiari District, Pakistan: A community-based cross-sectional study
Source: PLoS One. 2026 Jun 17;21(6):e0350609. doi: 10.1371/journal.pone.0350609 (PMC13274832; doi:10.1371/journal.pone.0350609)
Supplement: S2 Table — (DOCX) [file pone.0350609.s002.docx]

**S2 Table. Prevalence estimates in boys and girls, by age.**

|  |  | Boys (n=678) | | | | Girls (n=718) | | | |  |
| --- | --- | --- | --- | --- | --- | --- | --- | --- | --- | --- |
|  | Age (years) | N | Estimate | [95%CI] | | N | Estimate | [95%CI] | | p |
| **Depressive symptoms** | **All** | **678** | **8.11** | **[6.28-10.41]** | | **718** | **10.17** | **[8.16-12.59]** | |  |
| **(SMFQ score ≥ 8)** | 9 |  |  |  |  | 92 | 6.52 | [3.02-13.51] | |  |
|  | 10 | 115 | 6.96 | [3.57-13.13] | | 148 | 6.08 | [3.23-11.15] | | .775 |
|  | 11 | 104 | 7.69 | [3.95-14.45] | | 107 | 12.15 | [7.24-19.68] | | .280 |
|  | 12 | **143** | **5.59** | **[2.86-10.65]** | | **125** | **17.60** | **[11.92-25.21]** | | **.002** |
|  | 13 | 102 | 12.75 | [7.60-20.59] | | 130 | 13.85 | [8.94-20.83] | | .807 |
|  | 14 | 134 | 9.70 | [5.76-15.89] | | 116 | 4.31 | [1.85-9.69] | | .100 |
|  | 15 | 80 | 6.3 | [2.70-13.81] | |  |  |  |  |  |
| **Panic disorder or significant somatic disorder** | **All** | **678** | **13.27** | **[10.93-16.04]** | | **718** | **17.13** | **[14.55-20.06]** | |  |
| **(SCARED subscale score ≥ 7)** | 9 |  |  |  |  | 92 | 14.13 | [8.45-22.69] | |  |
|  | 10 | 115 | 13.04 | [0.81-20.41] | | 148 | 16.89 | [11.71-23.75] | | .389 |
|  | 11 | 104 | 18.27 | [12.02-26.78] | | 107 | 16.82 | [10.91-25.03] | | .782 |
|  | 12 | **143** | **11.19** | **[7.01-17.40]** | | **125** | **22.40** | **[15.98-30.47]** | | **.013** |
|  | 13 | 102 | 15.69 | [9.89-23.97] | | 130 | 20.00 | [14.03-27.69] | | .397 |
|  | 14 | 134 | 14.18 | [9.27-21.09] | | 116 | 11.21 | [6.67-18.23] | | .483 |
|  | 15 | 80 | 6.25 | [2.70-13.81] | |  |  |  |  |  |
| **Generalized anxiety disorder** | **All** | **678** | **5.75** | **[4.24-7.77]** | | **718** | **7.66** | **[5.93-9.84]** | |  |
| **(SCARED subscale score ≥ 9)** | 9 |  |  |  |  | 92 | 4.35 | [1.70-10.65] | |  |
|  | 10 | 115 | 4.35 | [1.87-9.78] | | 148 | 3.38 | [1.45-7.66] | | .683 |
|  | 11 | 104 | 4.81 | [2.07-10.76] | | 107 | 7.48 | [3.84-14.06] | | .420 |
|  | 12 | **143** | **5.59** | **[2.86-10.65]** | | **125** | **12.80** | **[8.03-19.78]** | | **.039** |
|  | 13 | 102 | 7.84 | [4.03-14.72] | | 130 | 10.77 | [6.52-17.27] | | .450 |
|  | 14 | 134 | 7.46 | [4.11-13.19] | | 116 | 6.90 | [3.54-13.02] | | .863 |
|  | 15 | 80 | 3.75 | [1.28-10.45] | |  |  |  |  |  |
| **Separation anxiety disorder** | **All** | **678** | **23.89** | **[20.84-27.25]** | | **718** | **39.14** | **[35.63-42.76]** | |  |
| **(SCARED subscale score ≥ 5)** | 9 |  |  |  |  | 92 | 44.57 | [34.83-54.74] | |  |
|  | 10 | 115 | 24.35 | [17.42-32.94] | | 148 | 33.78 | [26.66-41.73] | | .097 |
|  | 11 | **104** | **29.81** | **[21.86-39.19]** | | **107** | **45.79** | **[36.66-55.22]** | | **.017** |
|  | 12 | **143** | **27.27** | **[20.64-35.10]** | | **125** | **46.40** | **[37.80-55.12]** | | **.001** |
|  | 13 | **102** | **22.55** | **[15.52-31.57]** | | **130** | **37.69** | **[29.83-46.26]** | | **.013** |
|  | 14 | **134** | **18.66** | **[12.97-26.09]** | | **116** | **29.31** | **[21.80-38.15]** | | **.048** |
|  | 15 | 80 | 20.00 | [12.70-30.05] | |  |  |  |  |  |
| **Social anxiety disorder** | **All** | **678** | **11.21** | **[9.05-13.81]** | | **718** | **22.28** | **[19.39-25.47]** | |  |
| **(SCARED subscale score ≥ 8)** | 9 |  |  |  |  | 92 | 16.3 | [10.14-25.17] | |  |
|  | 10 | 115 | 14.78 | [9.44-22.40] | | 148 | 20.95 | [15.17-28.19] | | .199 |
|  | 11 | 104 | 12.50 | [7.45-20.22] | | 107 | 18.69 | [12.44-27.11] | | .216 |
|  | 12 | **143** | **13.99** | **[9.24-20.62]** | | **125** | **31.20** | **[23.74-39.78]** | | **.001** |
|  | 13 | **102** | **9.80** | **[5.41-17.11]** | | **130** | **27.69** | **[20.72-35.94]** | | **.001** |
|  | 14 | **134** | **7.46** | **[4.10-13.19]** | | **116** | **16.38** | **[10.74-24.17]** | | **.028** |
|  | 15 | 80 | 7.5 | [3.48-15.41] | |  |  |  |  |  |
| **School avoidance*** | **All** | **508** | **15.75** | **[12.84-19.17]** | | **375** | **15.47** | **[12.16-19.47]** | |  |
| **(SCARED subscale score ≥ 3)** | 9 |  |  |  |  | 70 | 17.14 | [10.09-27.62] | |  |
|  | 10 | 100 | 18.00 | [11.70-26.67] | | 97 | 12.37 | [7.22-20.39] | | .272 |
|  | 11 | 90 | 14.44 | [8.64-23.16] | | 59 | 16.95 | [9.48-28.46] | | .679 |
|  | 12 | 11 | 20.72 | [14.22-19.18] | | 61 | 21.31 | [12.90-33.12] | | .927 |
|  | 13 | 71 | 14.08 | [7.83-24.02] | | 46 | 15.22 | [7.57-28.22] | | .865 |
|  | 14 | 87 | 12.64 | [7.21-21.24] | | 42 | 9.52 | [3.77-22.07] | | .604 |
|  | 15 | 49 | 10.20 | [4.44-21.76] | |  |  |  |  |  |

Confidence Intervals obtained using the Wilson Score method. * In adolescents attending school; SMFQ: Short Mood and Feelings Questionnaire; SCARED: Screen for Child Anxiety Related Disorders.
