# Supplementary material for: Depression, anxiety symptoms, and association with household characteristics in adolescent boys and girls from Matiari District, Pakistan: A community-based cross-sectional study
Source: PLoS One. 2026 Jun 17;21(6):e0350609. doi: 10.1371/journal.pone.0350609 (PMC13274832; doi:10.1371/journal.pone.0350609)
Supplement: S3 Table — (DOCX) [file pone.0350609.s003.docx]

**S3 Table. Spearman correlations (ρ) between covariates in boys and girls**

|  | Age | Living Area | School Attendance | Mother's marital status | Mother's working status | Mother school attendance | Partner’s occupation | Partner’s school attendance | Intimate partner violence | Wealth Index | Food insecurity | Mother's mental health well-being |
| --- | --- | --- | --- | --- | --- | --- | --- | --- | --- | --- | --- | --- |
| **In Boys (n=678):** |  |  |  |  |  |  |  |  |  |  |  |  |
| Age | 1 |  |  |  |  |  |  |  |  |  |  |  |
| Living Area | -0.060 | 1 |  |  |  |  |  |  |  |  |  |  |
| School Attendance | 0.217 | -0.062 | 1 |  |  |  |  |  |  |  |  |  |
| Mother's marital status | 0.080 | -0.039 | 0.078 | 1 |  |  |  |  |  |  |  |  |
| Mother's working status | 0.031 | 0.082 | -0.155 | -0.034 | 1 |  |  |  |  |  |  |  |
| Mother school attendance | 0.037 | -0.058 | -0.105 | 0.018 | 0.109 | 1 |  |  |  |  |  |  |
| Partner’s occupation | 0.061 | -0.020 | -0.067 | -0.034 | 0.136 | 0.199 | 1 |  |  |  |  |  |
| Partner’s school attendance | -0.032 | 0.029 | -0.235 | -0.114 | 0.221 | 0.219 | 0.264 | 1 |  |  |  |  |
| Intimate partner violence | 0.036 | -0.049 | 0.024 | 0.474 | -0.004 | 0.032 | -0.074 | -0.021 | 1 |  |  |  |
| Wealth Index | -0.093 | -0.033 | 0.116 | 0.044 | -0.137 | -0.203 | -0.201 | -0.173 | 0.039 | 1 |  |  |
| Food insecurity | 0.075 | -0.071 | 0.140 | 0.139 | -0.198 | -0.137 | -0.128 | -0.151 | 0.054 | 0.181 | 1 |  |
| Mother's mental health well-being | 0.023 | 0.073 | -0.060 | -0.062 | 0.094 | 0.043 | 0.064 | 0.075 | -0.093 | -0.095 | -0.233 | 1 |
| **In Girls (n=718):** |  |  |  |  |  |  |  |  |  |  |  |  |
| Age | 1 |  |  |  |  |  |  |  |  |  |  |  |
| Living Area | 0.107 | 1 |  |  |  |  |  |  |  |  |  |  |
| School Attendance | 0.281 | 0.128 | 1 |  |  |  |  |  |  |  |  |  |
| Mother's marital status | 0.080 | -0.013 | 0.018 | 1 |  |  |  |  |  |  |  |  |
| Mother's working status | 0.026 | 0.025 | -0.153 | -0.130 | 1 |  |  |  |  |  |  |  |
| Mother school attendance | 0.029 | -0.091 | -0.346 | -0.037 | 0.139 | 1 |  |  |  |  |  |  |
| Partner’s occupation | -0.080 | -0.063 | -0.224 | -0.009 | 0.087 | 0.144 | 1 |  |  |  |  |  |
| Partner’s school attendance | -0.069 | -0.047 | -0.273 | 0.017 | 0.184 | 0.240 | 0.239 | 1 |  |  |  |  |
| Intimate partner violence | 0.069 | -0.018 | 0.012 | 0.516 | -0.033 | -0.021 | -0.077 | -0.032 | 1 |  |  |  |
| Wealth Index | -0.007 | 0.005 | 0.176 | 0.075 | -0.122 | -0.166 | -0.089 | -0.113 | 0.018 | 1 |  |  |
| Food insecurity | 0.106 | -0.058 | 0.200 | 0.151 | -0.214 | -0.121 | -0.133 | -0.128 | 0.100 | 0.183 | 1 |  |
| Mother's mental health well-being | -0.048 | 0.028 | -0.112 | -0.153 | 0.153 | 0.097 | 0.101 | 0.081 | -0.207 | -0.084 | -0.223 | 1 |
