# Supplementary material for: Depression, anxiety symptoms, and association with household characteristics in adolescent boys and girls from Matiari District, Pakistan: A community-based cross-sectional study
Source: PLoS One. 2026 Jun 17;21(6):e0350609. doi: 10.1371/journal.pone.0350609 (PMC13274832; doi:10.1371/journal.pone.0350609)
Supplement: S4 Table — (DOCX) [file pone.0350609.s004.docx]

**S4 Table: Association of household characteristics with depressive symptoms in boys living in Matiari, Pakistan (n=678).**

|  | | M1^a^ | | | | | |  | | | M2 | | | | | | | |  | | M3 | | | | | |
| --- | --- | --- | --- | --- | --- | --- | --- | --- | --- | --- | --- | --- | --- | --- | --- | --- | --- | --- | --- | --- | --- | --- | --- | --- | --- | --- |
|  | | IRR | | [95%CI] | | p | |  | | | IRR | | [95%CI] | | | | p | |  | | IRR | | [95%CI] | | | p |
| **Participants characteristics** | |  | |  |  |  | |  | | |  | |  | |  | |  | |  | |  | |  |  | |  |
| **Age*** | | 1.023 | | 0.956-1.094 | | .516 | |  | | | 1.011 | | 0.946-1.080 | | | | .754 | |  | | 1.044 | | 0.982-1.110 | | | .171 |
| **School attendance*** | |  | | 0.794-1.310 | |  | |  | | |  | |  | |  | |  | |  | |  | |  |  | |  |
| No | | 1.019 | |  |  | .880 | |  | | | 0.980 | | 0.766-1.254 | | | | .873 | |  | | 0.881 | | 0.700-1.109 | | | .280 |
| Yes | | Ref | |  |  |  | |  | | | Ref | |  | |  | |  | |  | | Ref | |  |  | |  |
|  | |  | |  |  |  | |  | | |  | |  | |  | |  | |  | |  | |  |  | |  |
| **Household characteristics** | |  | |  |  |  | |  | | |  | |  | |  | |  | |  | |  | |  |  | |  |
| **Living Area** | |  | |  |  |  | |  | | |  | |  | |  | |  | |  | |  | |  |  | |  |
| Urban | | Ref | |  |  |  | |  | | | Ref | |  | |  | |  | |  | | Ref | |  |  | |  |
| Rural | | 0.813 | | 0.622-1.063 | | .130 | |  | | | 0.852 | | 0.656-1.060 | | | | .228 | |  | | 0.822 | | 0.648-1.043 | | | .107 |
| **Mother's marital status** | |  | |  |  |  | |  | | |  | |  | |  | |  | |  | |  | |  |  | |  |
| Married | | Ref | |  |  |  | |  | | | Ref | |  | |  | |  | |  | | Ref | |  |  | |  |
| Widowed, divorced or separated | | 1.193 | | 0.783-1.818 | | .411 | |  | | | 1.033 | | 0.309-3.459 | | | | .958 | |  | | 1.042 | | 0.357-3.042 | | | .940 |
| **Mother's working status** | |  | |  |  |  | |  | | |  | |  | |  | |  | |  | |  | |  |  | |  |
| Working | | Ref | |  |  |  | |  | | | Ref | |  | |  | |  | |  | | Ref | |  |  | |  |
| Homemaker | | **0.713** | | **0.574-0.884** | | **.002** | |  | | | **0.775** | | **0.625-0.959** | | | | **.019** | |  | | **0.744** | | **0.611-0.906** | | | **.003** |
| **Mother school attendance** | |  | |  |  |  | |  | | |  | |  | |  | |  | |  | |  | |  |  | |  |
| No | | 0.998 | | 0.741-1.346 | | .991 | |  | | | 0.902 | | 0.666-1.221 | | | | .504 | |  | | 0.928 | | 0.701-1.227 | | | .600 |
| Yes | | Ref | |  |  |  | |  | | | Ref | |  | |  | |  | |  | | Ref | |  |  | |  |
| **Partner’s occupation** | |  | |  |  |  | |  | | |  | |  | |  | |  | |  | |  | |  |  | |  |
| Manual labour, agriculture | | Ref | |  |  |  | |  | | | Ref | |  | |  | |  | |  | | Ref | |  |  | |  |
| Sales, service, professional, others | | 0.893 | | 0.672-1.188 | | .438 | |  | | | 1.111 | | 0.821-1.503 | | | | .497 | |  | | 1.258 | | 0.951-1.663 | | | .108 |
| Unemployed | | 2.277 | | 0.856-6.059 | | .099 | |  | | | 2.327 | | 0.909-5.960 | | | | .078 | |  | | 1.814 | | 0.799-4.121 | | | .155 |
| **Partner’s school attendance** | |  | |  |  |  | |  | | |  | |  | |  | |  | |  | |  | |  |  | |  |
| No | | 1.080 | | 0.870-1.340 | | .485 | |  | | | 0.953 | | 0.764-1.189 | | | | .672 | |  | | 0.985 | | 0.803-1.208 | | | .881 |
| Yes | | Ref | |  |  |  | |  | | | Ref | |  | |  | |  | |  | | Ref | |  |  | |  |
|  | |  | |  |  |  | |  | | |  | |  | |  | |  | |  | |  | |  | (continues) | | |
| **Intimate partner violence against mother** | |  | |  |  |  | |  | | |  | |  | |  | |  | |  | |  | |  |  | |  |
| No | | Ref | |  |  |  | |  | | | Ref | |  | |  | |  | |  | | Ref | |  |  | |  |
| Yes | | **1.623** | | **1.283-2.054** | | **<.001** | |  | | | **1.667** | | **1.323-2.101** | | | | **<.001** | |  | | **1.614** | | **1.301-2.003** | | | **<.001** |
| Missing | | **1.678** | | **1.084-2.598** | | **.020** | |  | | | 1.357 | | 0.411-4.481 | | | | .617 | |  | | 1.143 | | 0.396-3.299 | | | .804 |
| **Wealth Index** | |  | |  |  |  | |  | | |  | | 0.718-1.118 | | | |  | |  | |  | |  |  | |  |
| Poor (Q1, Q2) | | 1.004 | | 0.807-1.248 | | .975 | |  | | | 0.896 | |  |  |  |  | .330 | |  | | 0.871 | | 0.710-1.070 | | | .188 |
| Non Poor (Q3, Q4, Q5) | | Ref | |  |  |  | |  | | | Ref | |  | |  | |  | |  | | Ref | |  |  | |  |
| **Food insecurity (FIES)** | |  | |  |  |  | |  | | |  | |  | |  | |  | |  | |  | |  |  | |  |
| Food secure/Mild food insecure | | Ref | |  |  |  | |  | | | Ref | |  | |  | |  | |  | | Ref | |  |  | |  |
| Moderate to severe food insecure | | **1.864** | | **1.475-2.356** | | **<.001** | |  | | | **1.899** | | **1.488-2.423** | | | | **<.001** | |  | | **1.454** | | **1.158-1.826** | | | **.001** |
| **Mother's mental health well-being** Score on the WEMWBS scale, mean [SD] | **0.947** | | **0.937-0.957** | | | | **<.001** | |  |  | |  | |  | |  | |  | | **0.950** | | **0.940-0.959** | | | **<.001** | |
| ^*^ Association estimated in the M0 Model (with the inclusions of age and school attendance only). ^a^ Adjusted for age and school attendance; IRR, Incidence Rate Ratio; CI, Confidence Interval; FIES, Food Insecurity Experience Scale; WEMWBS, Warwick-Edinburgh Mental Wellbeing Scale | | | | | | | | | | | | | | | | | | | | | | | | | | |
