# Supplementary material for: Depression, anxiety symptoms, and association with household characteristics in adolescent boys and girls from Matiari District, Pakistan: A community-based cross-sectional study
Source: PLoS One. 2026 Jun 17;21(6):e0350609. doi: 10.1371/journal.pone.0350609 (PMC13274832; doi:10.1371/journal.pone.0350609)
Supplement: S5 Table — (DOCX) [file pone.0350609.s005.docx]

**S5 Table. Association of household characteristics with anxiety symptoms in boys living in Matiari, Pakistan (n=678).**

|  | M1^a^ | | | |  | M1 | | | |  | M2 | | | |
| --- | --- | --- | --- | --- | --- | --- | --- | --- | --- | --- | --- | --- | --- | --- |
|  | IRR | [95%CI] | | p |  | IRR | [95%CI] | | p |  | IRR | [95%CI] | | p |
| **Participants characteristics** |  |  |  |  |  |  |  |  |  |  |  |  |  |  |
| Age* | 0.976 | 0.940-1.014 | | .218 |  | 0.974 | 0.938-1.011 | | .161 |  | 0.989 | 0.955-1.024 | | .525 |
| School attendance* |  |  |  |  |  |  |  |  |  |  |  |  |  |  |
| No | 0.912 | 0.791-1.051 | | .205 |  | 0.874 | 0.758-1.008 | | .064 |  | **0.845** | **0.740-0.966** | | **.014** |
| Yes | Ref |  |  |  |  | Ref |  |  |  |  | Ref |  |  |  |
|  |  |  |  |  |  |  |  |  |  |  |  |  |  |  |
| **Household characteristics** |  |  |  |  |  |  |  |  |  |  |  |  |  |  |
| Living Area |  |  |  |  |  |  |  |  |  |  |  |  |  |  |
| Urban | Ref |  |  |  |  | Ref |  |  |  |  | Ref |  |  |  |
| Rural | 0.936 | 0.803-1.091 | | .394 |  | 0.958 | 0.824-1.113 | | .572 |  | 0.960 | 0.835-1.103 | | .565 |
| Mother's marital status |  |  |  |  |  |  |  |  |  |  |  |  |  |  |
| Married | Ref |  |  |  |  | Ref |  |  |  |  | Ref |  |  |  |
| Widowed, divorced or separated | 0.884 | 0.694-1.127 | | .321 |  | 0.607 | 0.309-1.195 | | .149 |  | 0.634 | 0.340-1.181 | | .151 |
| Mother's working status |  |  |  |  |  |  |  |  |  |  |  |  |  |  |
| Working | Ref |  |  |  |  | Ref |  |  |  |  | Ref |  |  |  |
| Homemaker | **0.842** | **0.745**-**0.952** | | **.006** |  | 0.887 | 0.784-1.004 | | .058 |  | **0.891** | **0.794-0.999** | | **.047** |
| Mother school attendance |  |  |  |  |  |  |  |  |  |  |  |  |  |  |
| No | 1.150 | 0.972-1.360 | | .104 |  | 1.070 | 0.902-1.271 | | .437 |  | 1.075 | 0.916-1.261 | | .376 |
| Yes | Ref |  |  |  |  | Ref |  |  |  |  | Ref |  |  |  |
| Partner’s occupation |  |  |  |  |  |  |  |  |  |  |  |  |  |  |
| Manual labour, agriculture | Ref |  |  |  |  | Ref |  |  |  |  | Ref |  |  |  |
| Sales, service, professional, others | 0.905 | 0.810-1.115 | | .535 |  | 1.107 | 0.934-1.312 | | .240 |  | **1.176** | **1.005-1.376** | | **.043** |
| Unemployed | 1.406 | 0.785-2.519 | | .252 |  | 1.281 | 0.728-2.253 | | .391 |  | 1.106 | 0.656-1.864 | | .706 |
| Partner’s school attendance |  |  |  |  |  |  |  |  |  |  |  |  |  |  |
| No | 1.054 | 0.933-1.190 | | .400 |  | 1.010 | 0.890-1.147 | | .872 |  | 1.007 | 0.895-1.133 | | .906 |
| Yes | Ref |  |  |  |  | Ref |  |  |  |  | Ref |  |  |  |
|  |  |  |  |  |  |  |  |  |  |  |  |  | (continues) | |
| Intimate partner violence against mother |  |  |  |  |  |  |  |  |  |  |  |  |  |  |
| No | Ref |  |  |  |  | Ref |  |  |  |  | Ref |  |  |  |
| Yes | **1.226** | **1.076-1.398** | | **.002** |  | **1.235** | **1.086-1.404** | | **.001** |  | **1.198** | **1.062-1.350** | | **.003** |
| Missing | 1.077 | 0.838-1.384 | | .561 |  | 1.518 | 0.780-2.955 | | .219 |  | 1.313 | 0.712-2.424 | | .383 |
| Wealth Index |  |  |  |  |  |  |  |  |  |  |  |  |  |  |
| Poor (Q1, Q2) | 1.087 | 0.961-1.230 | | .183 |  | 1.007 | 0.888-1.142 | | .914 |  | 1.000 | 0.890-1.124 | | .995 |
| Non Poor (Q3, Q4, Q5) | Ref |  |  |  |  | Ref |  |  |  |  | Ref |  |  |  |
| Food insecurity (FIES) |  |  |  |  |  |  |  |  |  |  |  |  |  |  |
| Food secure/Mild food insecure | Ref |  |  |  |  | Ref |  |  |  |  | Ref |  |  |  |
| Moderate to severe food insecure | **1.433** | **1.251-1.642** | | **<.001** |  | **1.440** | **1.250-1.658** | | **<.001** |  | **1.224** | **1.070-1.401** | | **.003** |
| Mother's mental health well-being Score on the WEMWBS scale, mean [SD] | **0.968** | **0.962-0.974** | | **<.001** |  |  |  |  |  |  | **0.970** | **0.964-0.976** | | **<.001** |
| *Association estimated in the M0 Model (with the inclusions of age and school attendance only). ^a^ Adjusted for age and school attendance; IRR, Incidence Rate Ratio; CI, Confidence Interval; FIES, Food Insecurity Experience Scale; WEMWBS, Warwick-Edinburgh Mental Wellbeing Scale | | | | | | | | | | | | | | |
