# Supplementary material for: Depression, anxiety symptoms, and association with household characteristics in adolescent boys and girls from Matiari District, Pakistan: A community-based cross-sectional study
Source: PLoS One. 2026 Jun 17;21(6):e0350609. doi: 10.1371/journal.pone.0350609 (PMC13274832; doi:10.1371/journal.pone.0350609)
Supplement: S6 Table — (DOCX) [file pone.0350609.s006.docx]

**S6 Table. Association of household characteristics with depressive symptoms in girls living in Matiari, Pakistan (n=718).**

|  | M1^a^ | | | | |  | | M2 | | | |  | | | M3 | | | |  | | M3 + interaction | | | | | |
| --- | --- | --- | --- | --- | --- | --- | --- | --- | --- | --- | --- | --- | --- | --- | --- | --- | --- | --- | --- | --- | --- | --- | --- | --- | --- | --- |
|  | IRR | [95%CI] | p | | |  | IRR | [95%CI] | | p |  | | IRR | [95%CI] | | | p |  | | IRR | [95%CI] | | p | |  |  |
| **Participants characteristics** |  |  | |  |  |  |  |  |  |  |  | |  |  | |  |  |  | |  |  |  |  | |  |  |
| Age* | 1.001 | 0.941-1.065 | .970 | | |  | 1.003 | 0.944-1.066 | | .916 |  | | 1.013 | 0.958-1.071 | | | .646 |  | | 1.014 | 0.959-1.071 | | .624 | |  |  |
| School attendance* |  |  | |  |  |  |  |  |  |  |  | |  |  | |  |  |  | |  |  |  |  | |  |  |
| No | **1.354** | **1.115-1.644** | **.002** | | |  | 1.211 | 0.981-1.497 | | .075 |  | | 1.166 | 0.957-1.421 | | | .127 |  | | 1.162 | 0.955-1.414 | | .134 | |  |  |
| Yes | Ref |  | |  |  |  | Ref |  |  |  |  | | Ref |  | |  |  |  | | Ref |  |  |  | |  |  |
|  |  |  | |  |  |  |  |  |  |  |  | |  |  | |  |  |  | |  |  |  |  | |  |  |
| **Household characteristics** |  |  | |  |  |  |  |  |  |  |  | |  |  | |  |  |  | |  |  |  |  | |  |  |
| Living Area |  |  | |  |  |  |  |  |  |  |  | |  |  | |  |  |  | |  |  |  |  | |  |  |
| Urban | Ref |  | |  |  |  | Ref |  |  |  |  | | Ref |  | |  |  |  | | Ref |  |  |  | |  |  |
| Rural | 0.817 | 0.660-1.013 | .065 | | |  | 0.841 | 0.682-1.037 | | .105 |  | | 0.858 | 0.706-1.041 | | | .121 |  | | 0.859 | 0.708-1.042 | | .123 | |  |  |
| Mother's marital status |  |  | |  |  |  |  |  |  |  |  | |  |  | |  |  |  | |  |  |  |  | |  |  |
| Married | Ref |  | |  |  |  | Ref |  |  |  |  | | Ref |  | |  |  |  | | Ref |  |  |  | |  |  |
| Widowed, divorced or separated | **1.411** | **1.004-1.984** | **.047** | | |  | 1.973 | 0.307-12.703 | | .474 |  | | 1.578 | 0.295-8.441 | | | .594 |  | | 1.399 | 0.257-7.624 | | .698 | |  |  |
| Mother's working status |  |  | |  |  |  |  |  |  |  |  | |  |  | |  |  |  | |  |  |  |  | |  |  |
| Working | Ref |  | |  |  |  | Ref |  |  |  |  | | Ref |  | |  |  |  | | Ref |  |  |  | |  |  |
| Homemaker | **0.621** | **0.514-0.751** | **<.001** | | |  | **0.626** | **0.516-0.761** | | **<.001** |  | | **0.727** | **0.606-0.871** | | | **.001** |  | | **0.716** | **0.598-0.858** | | **<.001** | |  |  |
| Mother’s school attendance |  |  | |  |  |  |  |  |  |  |  | |  |  | |  |  |  | |  |  |  |  | |  |  |
| No | 1.010 | 0.789-1.292 | .936 | | |  | 0.922 | 0.723-1.175 | | .511 |  | | 0.850 | 0.678-1.067 | | | .161 |  | | 0.859 | 0.685-1.076 | | .185 | |  |  |
| Yes | Ref |  | |  |  |  | Ref |  |  |  |  | | Ref |  | |  |  |  | | Ref |  |  |  | |  |  |
| Partner’s occupation |  |  | |  |  |  |  |  |  |  |  | |  |  | |  |  |  | |  |  |  |  | |  |  |
| Manual labour, agriculture | Ref |  | |  |  |  | Ref |  |  |  |  | | Ref |  | |  |  |  | | Ref |  |  |  | |  |  |
| Sales, service, professional, others | 0.819 | 0.636-1.055 | .122 | | |  | 0.936 | 0.728-1.204 | | .608 |  | | 0.990 | 0.782-1.254 | | | .934 |  | | 0.970 | 0.767-1.228 | | .803 | |  |  |
| Unemployed | 0.909 | 0.349-2.373 | .846 | | |  | 0.764 | 0.301-1.940 | | .571 |  | | 0.828 | 0.351-1.953 | | | .666 |  | | 0.838 | 0.357-1.966 | | .685 | |  |  |
| Partner’s school attendance |  |  | |  |  |  |  |  |  |  |  | |  |  | |  |  |  | |  |  |  |  | |  |  |
| No | 1.117 | 0.915-1.362 | .277 | | |  | 1.008 | 0.827-1.230 | | .934 |  | | 0.976 | 0.812-1.174 | | | .799 |  | | 0.989 | 0.822-1.189 | | .903 | |  |  |
| Yes | Ref |  | |  |  |  | Ref |  |  |  |  | | Ref |  | |  |  |  | | Ref |  |  |  | |  |  |
|  |  |  | |  |  |  |  |  |  |  |  | |  |  | |  |  |  | |  |  |  |  | |  |  |
|  |  |  | |  |  |  |  |  |  |  |  | |  |  | |  |  |  | |  |  | (continues) | | |  |  |
|  |  |  | |  |  |  |  |  |  |  |  | |  |  | |  |  |  | |  |  |  |  | |  |  |
| Intimate partner violence against mother |  |  | |  |  |  |  |  |  |  |  | |  |  | |  |  |  | |  |  |  |  | |  |  |
| No | Ref |  | |  |  |  | Ref |  |  |  |  | | Ref |  | |  |  |  | | Ref |  |  |  | |  |  |
| Yes | **1.521** | **1.246-1.858** | **<.001** | | |  | **1.600** | **1.312-1.950** | | **<.001** |  | | **1.411** | **1.172-1.699** | | | **<.001** |  | | **1.421** | **1.181-1.709** | | **<.001** | |  |  |
| Missing | **1.774** | **1.239-2.542** | **.002** | | |  | 0.801 | 0.123-5.211 | | .817 |  | | 0.703 | 0.130-3.797 | | | .683 |  | | 0.784 | 0.143-4.310 | | .780 | |  |  |
| Wealth Index |  |  | |  |  |  |  |  |  |  |  | |  |  | |  |  |  | |  |  |  |  | |  |  |
| Poor (Q1, Q2) | 1.055 | 0.871-1.279 | 0.583 | | |  | 0.950 | 0.785-1.150 | | .599 |  | | 0.937 | 0.784-1.118 | | | .469 |  | | 0.935 | 0.783-1.115 | | .452 | |  |  |
| Non Poor (Q3, Q4, Q5) | Ref |  | |  |  |  | Ref |  |  |  |  | | Ref |  | |  |  |  | | Ref |  |  |  | |  |  |
| Food insecurity (FIES) |  |  | |  |  |  |  |  |  |  |  | |  |  | |  |  |  | |  |  |  |  | |  |  |
| Food secure/Mild food insecure | Ref |  | |  |  |  | Ref |  |  |  |  | | Ref |  | |  |  |  | | Ref |  |  |  | |  |  |
| Moderate to severe food insecure | **1.508** | **1.210-1.878** | **<.001** | | |  | **1.352** | **1.084-1.687** | | **.008** |  | | 1.066 | 0.864-1.314 | | | .553 |  | | **3.040** | **1.195-7.735** | | **.020** | |  |  |
| Mother's mental health well-being Score on the WEMWBS scale, mean [SD] | **0.952** | **0.944-0.960** | **<.001** | | |  |  |  |  |  |  | | **0.956** | **0.948-0.965** | | | **<.001** |  | | **0.962** | **0.953-0.972** | | **<.001** | |  |  |
| Mother's mental health well-being X Food insecurity |  |  | |  |  |  |  |  |  |  |  | |  |  | |  |  |  | | **0.978** | **0.959-0.997** | | **.024** | |  |  |
| *Association estimated in the M0 Model (with the inclusions of age and school attendance only). ^a^ Adjusted for age and school attendance; IRR, Incidence Rate Ratio; CI, Confidence Interval; FIES, Food Insecurity Experience Scale; WEMWBS, Warwick-Edinburgh Mental Wellbeing Scale | | | | | | | | | | | | | | | | | | | | | | | |  | |  |
