# Supplementary material for: Depression, anxiety symptoms, and association with household characteristics in adolescent boys and girls from Matiari District, Pakistan: A community-based cross-sectional study
Source: PLoS One. 2026 Jun 17;21(6):e0350609. doi: 10.1371/journal.pone.0350609 (PMC13274832; doi:10.1371/journal.pone.0350609)
Supplement: S7 Table — (DOCX) [file pone.0350609.s007.docx]

**S7 Table. Association of household characteristics with anxiety symptoms in girls living in Matiari, Pakistan (n=718).**

|  | M1^a^ | | | |  | M2 | | | |  | M3 | | | |
| --- | --- | --- | --- | --- | --- | --- | --- | --- | --- | --- | --- | --- | --- | --- |
|  | IRR | [95%CI] | | p |  | IRR | [95%CI] | | p |  | IRR | [95%CI] | | p |
| **Participants characteristics** |  |  |  |  |  |  |  |  |  |  |  |  |  |  |
| Age* | 0.990 | 0.958 | 1.023 | 0.544 |  | 0.990 | 0.958 | 1.022 | 0.531 |  | 0.990 | 0.961 | 1.020 | 0.498 |
| School attendance* |  |  |  |  |  |  |  |  |  |  |  |  |  |  |
| No | **1.196** | **1.075** | **1.330** | **0.001** |  | **1.126** | **1.003** | **1.263** | **0.044** |  | 1.104 | 0.991 | 1.230 | 0.073 |
| Yes | Ref |  |  |  |  | Ref |  |  |  |  | Ref |  |  |  |
|  |  |  |  |  |  |  |  |  |  |  |  |  |  |  |
| **Household characteristics** |  |  |  |  |  |  |  |  |  |  |  |  |  |  |
| Living Area |  |  |  |  |  |  |  |  |  |  |  |  |  |  |
| Urban | Ref |  |  |  |  | Ref |  |  |  |  | Ref |  |  |  |
| Rural | **0.867** | **0.771** | **0.975** | **0.017** |  | **0.883** | **0.787** | **0.991** | **0.035** |  | **0.882** | **0.792** | **0.982** | **0.022** |
| Mother's marital status |  |  |  |  |  |  |  |  |  |  |  |  |  |  |
| Married | Ref |  |  |  |  | Ref |  |  |  |  | Ref |  |  |  |
| Widowed, divorced or separated | 1.144 | 0.945 | 1.384 | 0.167 |  | 0.990 | 0.386 | 2.535 | 0.983 |  | 0.856 | 0.349 | 2.102 | 0.735 |
| Mother's working status |  |  |  |  |  |  |  |  |  |  |  |  |  |  |
| Working | Ref |  |  |  |  | Ref |  |  |  |  | Ref |  |  |  |
| Homemaker | **0.819** | **0.738** | **0.910** | **<0.001** |  | **0.847** | **0.761** | **0.943** | **0.002** |  | **0.897** | **0.811** | **0.992** | **0.034** |
| Mother’s school attendance |  |  |  |  |  |  |  |  |  |  |  |  |  |  |
| No | 1.040 | 0.911 | 1.188 | 0.562 |  | 1.017 | 0.891 | 1.159 | 0.807 |  | 0.985 | 0.871 | 1.114 | 0.813 |
| Yes | Ref |  |  |  |  | Ref |  |  |  |  | Ref |  |  |  |
| Partner’s occupation |  |  |  |  |  |  |  |  |  |  |  |  |  |  |
| Manual labour, agriculture | Ref |  |  |  |  | Ref |  |  |  |  | Ref |  |  |  |
| Sales, service, professional, others | 0.942 | 0.823 | 1.079 | 0.391 |  | 1.006 | 0.879 | 1.151 | 0.936 |  | 1.040 | 0.916 | 1.180 | 0.546 |
| Unemployed | 0.968 | 0.576 | 1.628 | 0.903 |  | 0.924 | 0.557 | 1.532 | 0.758 |  | 0.945 | 0.590 | 1.513 | 0.813 |
| Partner’s school attendance |  |  |  |  |  |  |  |  |  |  |  |  |  |  |
| No | 1.050 | 0.943 | 1.169 | 0.370 |  | 1.004 | 0.901 | 1.118 | 0.948 |  | 1.011 | 0.914 | 1.119 | 0.829 |
| Yes | Ref |  |  |  |  | Ref |  |  |  |  | Ref |  |  |  |
|  |  |  |  |  |  |  |  |  |  |  |  |  | (continues) | |
| Intimate partner violence against mother |  |  |  |  |  |  |  |  |  |  |  |  |  |  |
| No | Ref |  |  |  |  | Ref |  |  |  |  | Ref |  |  |  |
| Yes | **1.252** | **1.124** | **1.394** | **<0.001** |  | **1.260** | **1.133** | **1.400** | **<0.001** |  | **1.176** | **1.064** | **1.299** | **0.001** |
| Missing | **1.306** | **1.069** | **1.594** | **0.009** |  | 1.170 | 0.456 | 3.003 | 0.744 |  | 1.118 | 0.455 | 2.747 | 0.808 |
| Wealth Index |  |  |  |  |  |  |  |  |  |  |  |  |  |  |
| Poor (Q1, Q2) | 1.037 | 0.934 | 1.151 | 0.499 |  | 0.981 | 0.885 | 1.089 | 0.721 |  | 0.957 | 0.869 | 1.055 | 0.376 |
| Non Poor (Q3, Q4, Q5) | Ref |  |  |  |  | Ref |  |  |  |  | Ref |  |  |  |
| Food insecurity (FIES) |  |  |  |  |  |  |  |  |  |  |  |  |  |  |
| Food secure/Mild food insecure | Ref |  |  |  |  | Ref |  |  |  |  | Ref |  |  |  |
| Moderate to severe food insecure | **1.335** | **1.184** | **1.506** | **<0.001** |  | **1.276** | **1.127** | **1.445** | **<0.001** |  | **1.138** | **1.011** | **1.280** | **0.032** |
| Mother's mental health well-being Score on the WEMWBS scale, mean [SD] | **0.973** | **0.969** | **0.978** | **<0.001** |  |  |  |  |  |  | **0.975** | **0.970** | **0.980** | **<0.001** |
| *Association estimated in the M0 Model (with the inclusions of age and school attendance only). ^a^ Adjusted for age and school attendance; IRR, Incidence Rate Ratio; CI, Confidence Interval; FIES, Food Insecurity Experience Scale; WEMWBS, Warwick-Edinburgh Mental Wellbeing Scale | | | | | | | | | | | | | | |
